# Early and multiple doses of zoledronate mitigates rebound bone loss following withdrawal of receptor activator of nuclear factor kappa-B ligand inhibition

**DOI:** 10.1093/jbmr/zjaf008

**Published:** 2025-01-23

**Authors:** Albert S Kim, Victoria E Taylor, Ariel Castro-Martinez, Suraj Dhakal, Amjad Zamerli, Sindhu T Mohanty, Ya Xiao, Marija K Simic, Alyssa Pantalone, Julian Chu, Tegan L Cheng, Peter I Croucher, Jacqueline R Center, Christian M Girgis, Michelle M McDonald

**Affiliations:** Cancer Ecosystems Program, Garvan Institute of Medical Research, Sydney, NSW 2010, Australia; Faculty of Medicine and Health, St Vincent’s Clinical School, UNSW Sydney, Sydney, NSW 2010, Australia; Faculty of Medicine and Health, University of Sydney, Sydney, NSW 2006, Australia; Department of Diabetes and Endocrinology, Westmead Hospital, Sydney, NSW 2145, Australia; Cancer Ecosystems Program, Garvan Institute of Medical Research, Sydney, NSW 2010, Australia; Faculty of Medicine and Health, University of Sydney, Sydney, NSW 2006, Australia; Cancer Ecosystems Program, Garvan Institute of Medical Research, Sydney, NSW 2010, Australia; Cancer Plasticity and Dormancy Program, Garvan Institute of Medical Research, Sydney, NSW 2010, Australia; Cancer Ecosystems Program, Garvan Institute of Medical Research, Sydney, NSW 2010, Australia; Cancer Ecosystems Program, Garvan Institute of Medical Research, Sydney, NSW 2010, Australia; Cancer Ecosystems Program, Garvan Institute of Medical Research, Sydney, NSW 2010, Australia; Cancer Ecosystems Program, Garvan Institute of Medical Research, Sydney, NSW 2010, Australia; Cancer Ecosystems Program, Garvan Institute of Medical Research, Sydney, NSW 2010, Australia; Department of Pathology, New York University Grossman School of Medicine, New York, NY 10016, United States; Faculty of Medicine and Health, University of Sydney, Sydney, NSW 2006, Australia; Centre for Children's Bone and Musculoskeletal Health, The Children's Hospital at Westmead, Sydney, NSW 2145, Australia; Faculty of Medicine and Health, University of Sydney, Sydney, NSW 2006, Australia; Centre for Children's Bone and Musculoskeletal Health, The Children's Hospital at Westmead, Sydney, NSW 2145, Australia; Faculty of Medicine and Health, St Vincent’s Clinical School, UNSW Sydney, Sydney, NSW 2010, Australia; Cancer Plasticity and Dormancy Program, Garvan Institute of Medical Research, Sydney, NSW 2010, Australia; Faculty of Medicine and Health, St Vincent’s Clinical School, UNSW Sydney, Sydney, NSW 2010, Australia; Cancer Plasticity and Dormancy Program, Garvan Institute of Medical Research, Sydney, NSW 2010, Australia; Faculty of Medicine and Health, University of Sydney, Sydney, NSW 2006, Australia; Department of Diabetes and Endocrinology, Westmead Hospital, Sydney, NSW 2145, Australia; Cancer Ecosystems Program, Garvan Institute of Medical Research, Sydney, NSW 2010, Australia; Faculty of Medicine and Health, St Vincent’s Clinical School, UNSW Sydney, Sydney, NSW 2010, Australia; Faculty of Medicine and Health, University of Sydney, Sydney, NSW 2006, Australia

**Keywords:** sequential therapy, zoledronate, denosumab, osteoporosis, denosumab discontinuation

## Abstract

Rebound bone loss following denosumab discontinuation is an important barrier in the effective long-term treatment of skeletal disorders. This is driven by increased osteoclastic bone resorption following the offset of RANKL inhibition, and sequential osteoclast-directed therapy has been utilized to mitigate this. However, current sequential treatment strategies intervene following the offset of RANKL inhibition and this approach fails to consistently prevent bone loss. Our previous work, using a mouse model of denosumab discontinuation, has shown that the processes that drive the rebound phenomenon occur earlier than when bone loss is detected, namely a rise and overshoot in serum tartrate-resistant acid phosphatase (TRAP). We identified that these changes in serum TRAP may provide an earlier window of opportunity to intervene with sequential therapy following RANKL inhibition withdrawal. Here, we show that early treatment with zoledronate (10 mg/kg, 3 wk following the last dose of OPG:Fc), preceding the rise and overshoot in serum TRAP, effectively mitigates rebound bone density loss through preventing the overshoot in serum TRAP. Further, we show that multiple doses of zoledronate (early treatment and during anticipated BMD loss) is superior in consolidating bone density gains made with RANKL inhibition and preventing rebound BMD loss as measured by DXA. Importantly, we demonstrate the efficacy of early and multi-dose zoledronate strategy in preventing bone loss in both growing and skeletally mature mice. MicroCT analysis showed improved trabecular bone structure in both the femur and lumbar vertebrae with zoledronate treatment compared with control. These increases in bone mass translated to increased fracture resistance in skeletally mature mice. This work provides a novel approach of early and multi-dose sequential treatment strategy following withdrawal of RANKL inhibition, contributing valuable insight into the clinical management of patients who discontinue denosumab therapy.

## Introduction

Neutralization of RANKL with denosumab has revolutionized the management of conditions requiring inhibition of bone resorption.^1^ Continued use leads to sustained BMD)gains and fracture risk reduction, with evidence of efficacy for up to 10 yr.^2^ Denosumab is well tolerated and often preferred by patients over bisphosphonates,^3^ leading to widespread use globally.

Rare, but serious side effects, such as medication-related osteonecrosis of the jaw (MRONJ) and atypical femoral fractures (AFFs), arise from prolonged suppression of bone turnover with antiresorptive therapy. This led to the development of “drug holidays” (or monitored treatment break),^4^ which reduces the risk of these complications in those receiving long term bisphosphonates.^5^ However, “drug holidays” are not recommended with denosumab. Bisphosphonates are embedded into bone tissue, displaying durability of osteoclast inhibition,^4^ whereas denosumab binds systemically to soluble and membrane bound RANKL, and osteoclast inhibition is rapidly reversed following its clearance.

Upon stopping denosumab, rebound BMD losses are observed with a sharp rise and overshoot in the markers of bone turnover following the offset of RANKL inhibition.^6^ Rise in P1NP and CTX above pretreatment baseline levels are observed from 6 mo following the last denosumab dose, following the offset of RANKL inhibition. These elevated levels of bone turnover markers are sustained for 12 mo before returning to baseline levels and are associated with increased fracture risk during this time, particularly the risk of multiple vertebral fractures.^7^ Despite these risks, persistence and adherence to denosumab declines with longer treatment duration.^8^

Rebound increases in bone resorption following denosumab discontinuation are driven by a rapid overshoot in osteoclastic activity.^9^ Therefore, an appropriate approach would be to transition to an osteoclast-directed therapy, such as bisphosphonates. However, clinical studies examining the use of sequential zoledronate do not consistently prevent rebound bone loss, with longer denosumab treatment duration and greater BMD gains being important risk factors for bone loss.^10–14^

Initial reports of rebound bone loss despite a “parting” dose of zoledronate^12^ gave rise to the idea that sequential therapy should be withheld until bone turnover resumes (as measured by serum CTX). It was thought that this would allow bisphosphonates to be taken up in the newly opened resorption pits and thereby become available to osteoclasts.^12^

However, prospective randomized controlled studies utilizing sequential zoledronate following denosumab discontinuation could not show consistent prevention of bone loss and fractures.^13–16^ Despite these inconclusive studies, current expert advice^17^ align sequential zoledronate with a rise in serum CTX or when BMD loss is detected, and while repeated zoledronate doses are recommended,^17^ the specific parameters that warrant this are not clearly defined, leading to bone loss and fractures despite multiple doses of zoledronate.^15^ There is currently no evidence-based, efficacious sequential treatment strategy to consolidate BMD gained on denosumab treatment, mitigate the rebound phenomenon, and prevent resultant fractures. This absence of a safe approach is rendering patients to receive denosumab indefinitely, and importantly, hindering treatment uptake.

An improved sequential treatment strategy following denosumab discontinuation is needed—based on better understanding of the cellular mechanisms driving this phenomenon and improved methods to predict imminent bone loss. Clinical studies examining sequential therapies are observational, and placebo-controlled studies are not ethically feasible due to the increased risk of bone loss and fractures in those receiving placebo.

**Figure 1:**
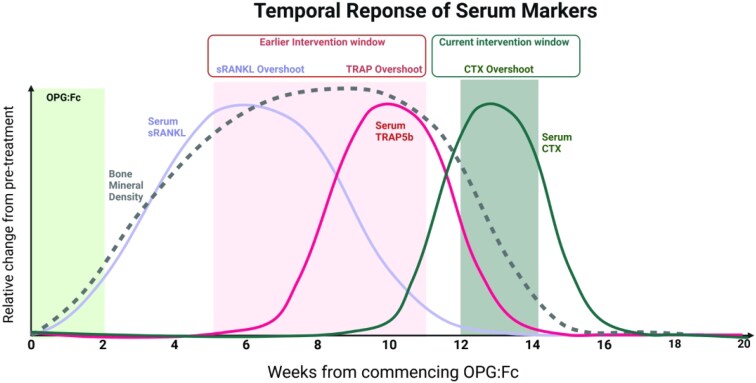
Pro-osteoclastogenic environment develops before a rise in CTX or BMD loss is detected following withdrawal of RANKL inhibition. Schematic summarizing our model of denosumab discontinuation utilizing OPG:Fc in growing mice showing the rebound phenomenon following withdrawal of RANKL inhibition.^18^ BMD and serum RANKL rise with OPG:Fc treatment, while serum TRAP and CTX are suppressed. As there is offset of RANKL inhibition, serum TRAP rises as BMD falls with a rise in CTX following this. Current sequential therapy strategies intervene at a time of CTX rise and/or BMD decline, at or later than 6 mo following the last denosumab dose, despite increased markers of osteoclast formation and activity preceding this. A rise in serum RANKL and TRAP may provide an earlier intervention window to prevent BMD loss analogous to within 6 mo of the last denosumab dose. Created in Biorender.com.

To address these limitations, we developed a murine model of denosumab discontinuation using osteoprotegerin(OPG:Fc), to mimic denosumab. In brief, OPG:Fc treatment leads to BMD gains and following the offset of its effect, there is rebound bone loss to vehicle levels. We showed that serum tartrate-resistant acid phosphatase (TRAP), a marker of osteoclast number and activity, rises prior to the rise in serum CTX and loss in BMD. We also revealed that a pro-osteoclastogenic environment develops before bone loss is clinically detected, highlighting a potential early intervention window with sequential therapy analogous to a time within 6 mo of the last denosumab dose^18^ (Figure 1). These findings suggest that rising serum TRAP may predict imminent bone loss and therefore support an optimized therapeutic window for sequential therapy, rather than waiting until bone loss is underway.

In this study, we first aimed to determine whether a single dose of zoledronate in the current therapeutic window, when CTX levels are rising and BMD loss underway, would be ineffective in preventing bone loss following denosumab discontinuation. We hypothesized that earlier intervention with bisphosphonates, prior to the offset of RANKL inhibition, and preceding the rise and overshoot of serum TRAP, would prevent rebound bone loss. We also aimed to determine whether a multi-dose zoledronate strategy, both early and a follow-up dose, would provide a more efficacious regimen for preventing rebound BMD loss. To translate the findings of our model to the clinical context, we performed these studies in both growing and skeletally mature mice to examine the efficacy of this strategy at various stages of skeletal maturity. Taken together, our preclinical studies provide novel insights to guide sequential treatment strategies in patients undertaking denosumab discontinuation.

## Materials and methods

### Experimental mice

Animal experiments were performed in accordance with approved protocols from the Garvan Institute of Medical Research and St Vincent’s Hospital Animal Ethics Committee(ARA 18/03 and 21/17), as well as the Australian Code of Practice for the Care and Use of Animals for Scientific Purposes.

Female C57BL/6J or C57BL/KaLwRij (Harlan, Netherlands) mice were obtained from Australian BioResources. All mice were bred and maintained under specific-pathogen free (SPF) conditions. Animal experiments were performed at the Biological Testing Facility, Garvan Institute of Medical Research. Holding areas were maintained within a constant temperature of 21.4 °C with humidity range to avoid stress and minimize experimental variability. Circadian rhythms were stimulated with lighting mimicking 12 h day/night cycles. Mice were provided with standard chow and water ad libitum.

All mice entered their respective experiments aged 6-8 or 26 wk and group sizes were determined based on previous experiences with each model, in which power calculations were performed to estimate sample size. Using this, 7-10 mice were allocated to each group or otherwise as stated in the figure legends.

To examine the effects of sequential zoledronate following OPG:Fc withdrawal modeling current clinical practice, C57BL/KaLwRij mice were randomly allocated to receive zoledronate or saline at week 12, 10 wk following 2 wk of OPG:Fc or saline treatment, at a time where a rise in serum CTX and rebound BMD loss was anticipated based on previous studies.^18^

To examine and compare the effects of early sequential zoledronate and a multi-dose zoledronate strategy following OPG:Fc withdrawal in young growing mice, 6-7 wk old C57BL/6J mice were randomly allocated to receive OPG:Fc or saline for 2 wk followed by zoledronate or saline at week 5 only, or week 5 and again at week 12 where CTX rise and BMD loss was anticipated in mice treated with OPG:Fc only.

To examine and compare the effects of early sequential zoledronate and a multi-dose zoledronate strategy following OPG:Fc withdrawal in skeletally mature mice, 26 wk old C57BL/6J mice were randomly allocated to receive OPG:Fc or saline for 4 wk, which was followed by zoledronate or saline at week 7 only, or week 7 and again at week 13.

Mice underwent DXA imaging and retro-orbital bleeds fortnightly or 3-weekly throughout the study. At the end of the study, tissue was harvested for their respective experiments outlined below.

### OPG:Fc and zoledronate treatment

OPG:Fc (Amgen Inc.) was administered at a dose of 10 mg/kg i.p. twice weekly for 2 wk in young growing mice (6-8 wk old). OPG:Fc was administered 3 times weekly for 4 wk in aged, skeletally mature mice (26 wk old) at a higher dose and for a longer duration to achieve a significant difference between vehicle and OPG:Fc-treated mice. OPG:Fc was administered at a dose confirmed to ablate osteoclasts.^19^ Vehicle mice received saline 2-3 times weekly at the same frequency as the OPG:Fc doses in the corresponding treatment groups.

Zoledronate(Novartis Pharma) was administered at a dose of 0.1 mg/kg i.p. once or repeated as outlined above, at a dose determined based on the adult therapeutic dose and confirmed to increase BMD in our previous work.^20^

### DXA analysis of BMD

DXA (Faxitron Ultrafocus DXA, Hologic) was performed fortnightly or 3-weekly on anesthetized mice under 3%-5% inhaled isoflurane. Hindlimb analysis was performed using VisionDXA (Hologic) and a manually drawn ROI encompassing the hindlimb excluding the foot was used to quantify BMD.

### MicroCT

Formalin-fixed right femora and the L4 vertebrae were imaged with the SkyScan 1772 microCT scanner (Bruker) at a resolution of 4.3 μm, 0.5 mm aluminum filter, 50 kv voltage, and 200 μA tube current. Images were captured every 0.4^o^ through 360^o^ and were reconstructed and analyzed using NRecon software (SkyScan). Bone structural parameters and nomenclature were utilized according to standardized guidelines.^21^ Three-dimensional reconstructed images of femora were generated using Drishti imaging software version 2.4 (ANU). ROI selection and analyses were performed using CTAn software (Bruker).

To examine the changes in femoral bone parameters trabecular bone parameters were calculated from scans performed at a voxel resolution of 5 μm in a 1 mm region beginning 200 μm proximal to the distal femoral growth plate to reduce the contribution of the primary spongiosa in the analysis. Cortical bone parameters were calculated from scans performed at a voxel resolution of 5 μm in a 0.5 mm region beginning 300 μm proximal to the distal femoral growth plate.

Changes in vertebral bone trabecular and cortical parameters were calculated from scans performed at a voxel resolution of 5 μm in an ROI calculated by measuring the distance between 0.2 mm offset from the point of 50% spongiosa and trabecular bone on both ends of the vertebral body.

### Measurement of TRAP5b and CTX

Serum collected by retro-orbital bleeds throughout animal phases, under anesthesia with isoflurane, was stored at −70° and then assessed for TRAP5b and CTX levels using ELISA kits (Immunodiagnostic Systems) following the manufacturer’s instructions.

### Mechanical testing

Compression testing of L4 vertebrae performed as described previously.^22^ In brief, the L4 vertebrae were warmed to room temperature and the vertebral processes were removed. Samples underwent mechanical testing on an Instron 5966 (Instron Inc.) by compression until failure. Testing was performed at 3 mm/min until breaking with a 100 N load cell. Data were collected using BlueHill 3 version 3 (Instron Inc.) and the load displacement curve were plotted with the maximum load to first failure calculated.

## Analysis of bone histomorphometry

### Quantitative histomorphometry

Preparation of femora samples for paraffin histomorphometric analysis and TRAP staining was performed as described previously.^18^ Briefly, femora were fixed in paraformaldehyde for 24 h then decalcified in EDTA. Samples were then processed in paraffin and sections were cut and stained for TRAP to identify osteoclasts from other resident bone cells.^18^

Right femora sections were scanned on the Aperio Scanscope CS2 model. An area of interest was indicated by a rectangle placed on the Macro image. Utilizing an Olympus UPLXAPO objective lens at a 20x objective, high-quality digital slides were created. Digital slides were modified on Aperio ImageScope (v12.3.2.8013) to show 3 ROI’s: 900 μm at 2.2x objective, 300 μm at 9.8x objective, and 300 μm at 7x objective.

Quantification of osteoclast populations among the trabecular bone were performed with BioQuant Osteo (Version v21.5.60). Utilizing a Zeiss Axioplan Microscope (Zeiss) with a high-resolution Jenoptik Camera at 10X objective, an ROI capturing a 3 mm region of trabecular bone, 1 mm from the top of the growth plate junction within the cortices was analyzed for each sample. TRAP-positive osteoclasts (bright pink cytoplasmic appearance) were marked, and their cell surface-bone contact perimeters and trabecular bone surfaces were recorded. The total bone surface, number of osteoclasts per total bone surface, and osteoclasts per total bone surface were determined. The structural and cellular parameters were calculated and expressed according to the ASBMR standardized nomenclature.^21^

### Statistical methods

Results were analyzed using GraphPad Prism (Version 9, GraphPad Prism). One-way ANOVA and multiple comparisons were performed using Tukey correction, and unpaired t-tests were performed when comparing 2 populations. All data are expressed as mean with error bars representing standard deviation and *p*-values less than .05 were considered statistically significant.

## Results

### Sequential zoledronate at the time of CTX rise does not prevent bone loss

**Figure 2:**
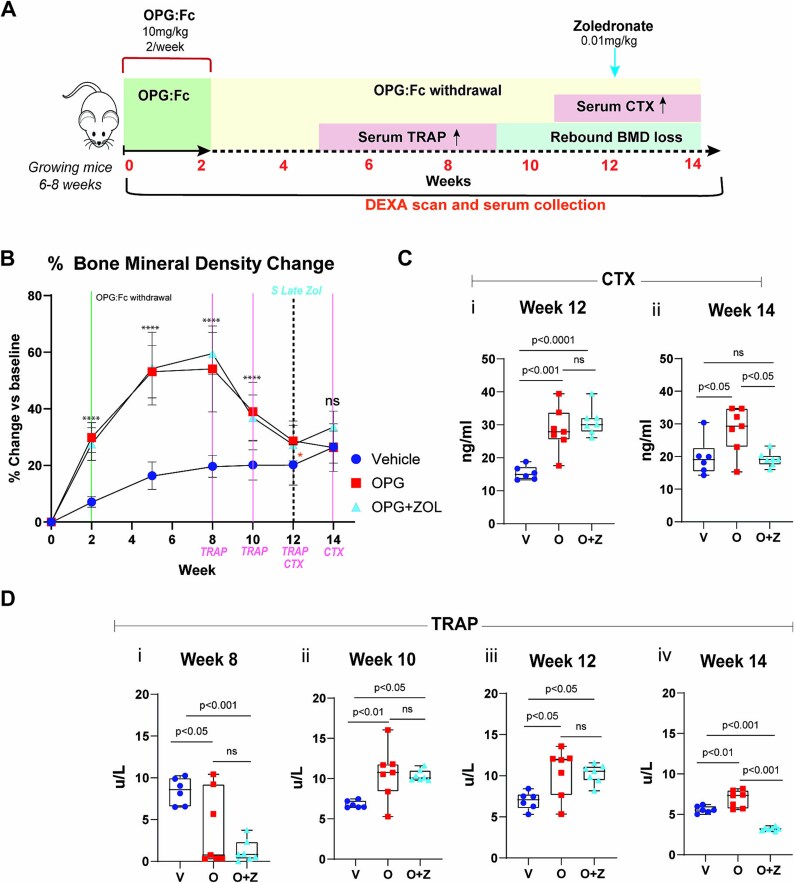
Sequential zoledronate at the time of CTX rise does not prevent bone loss. (A) Schematic of the experimental design to assess the effect of sequential zoledronate following OPG:Fc treatment in growing mice. Expected timing of a rise in serum TRAP, P1NP, CTX, and rebound bone loss highlighted. (B) BMD changes following OPG:Fc treatment and sequential treatment at week 12, at a time of expected CTX rise. BMD shown as percentage change from baseline levels following treatment with OPG:Fc or saline followed by zoledronate or saline at week 12 (*n* = 6 in vehicle, *n* = 7 per intervention group). Timing of zoledronate is denoted by a vertical dotted line at week 12. The vertical lines at weeks 8, 10, 12, and 14 denote time of CTX and TRAP analysis. Data are represented as mean ± SD. The asterisks indicate *p*-values <.05 (^*^p < .05, ^**^p < .01, ^****^p < .0001). (C) Serum CTX measured by ELISA at (i) week 12 and (ii) week 14. Data are represented as mean ± SD. (D) Serum TRAP measured by ELISA at (i) week 8, (ii) 10, (iii) 12, and (iv) 14. Data are represented as mean ± SD.

Serum CTX rise or BMD reduction has been used to guide the timing of sequential treatment^13^ due to concerns that earlier bisphosphonate administration would not be taken up onto bone surfaces.^12^ To model the current clinical practice, we treated mice with OPG:Fc or saline for 2 wk and administered zoledronate or saline at week 12, a time in our model of expected CTX rise from post treatment levels (Figure 2A).

Treatment with OPG:Fc (10 mg/kg) twice weekly for 2 wk significantly increased hindlimb BMD, which continued to rise for 6 wk following treatment reaching levels 21% higher than vehicle (*p* < .0001) before declining to vehicle levels over 4 wk between week 8 and 12. Zoledronate (0.1 mg/kg) or saline was administered at week 12, although BMD was equivalent between groups at this time point as rebound BMD loss had already occurred (Figure 2B).

Serum TRAP was 54% and 85% lower than vehicle at week 8 in mice treated with OPG:Fc (*p* < .05 and *p* < .001 in OPG and OPG + ZOL groups, respectively), though levels were rising from being fully suppressed following OPG:Fc treatment, indicating increasing osteoclast activity (Figure 2Di). Serum TRAP in OPG:Fc treated mice was 56% and 53% higher than vehicle at week 10 (*p* < .01 and *p* < .05, respectively, Figure 2Dii) and both serum TRAP and CTX were significantly higher than vehicle at week 12, when zoledronate was administered (Figures 2Ci and 2Diii). This elevation in TRAP and CTX above vehicle levels persisted in mice that received OPG:Fc-only at week 14, whereas administration of zoledronate at week 12 led to suppression of TRAP levels to significantly below vehicle levels (*p* < .001 OPG + ZOL vs Vehicle, Figure 2Div), and serum CTX to vehicle levels (Figure 2Cii), indicating reduced osteoclast activity.

These data confirm that the current intervention timing of sequential therapy, at a time of CTX rise or BMD loss, was too late to prevent rebound BMD loss following the offset of RANKL inhibition. This indicates that sequential bisphosphonate therapy may be more effective if administered before CTX levels rise, as our model showed that this is preceded by a rise in serum TRAP.

### Early and multiple sequential zoledronate consolidates BMD attained with RANKL inhibition and prevents the overshoot in serum TRAP

Our previous work showed that the processes that drive the rebound phenomenon occur before BMD loss is detected.^18^ We hypothesized that early intervention with zoledronate to prevent the rise and overshoot in serum TRAP would be able to prevent rebound BMD loss. Further we hypothesized that a repeated zoledronate dose at week 12, where we would expect rebound BMD loss based on our model, would be superior to the single early dose.

**Figure 3:**
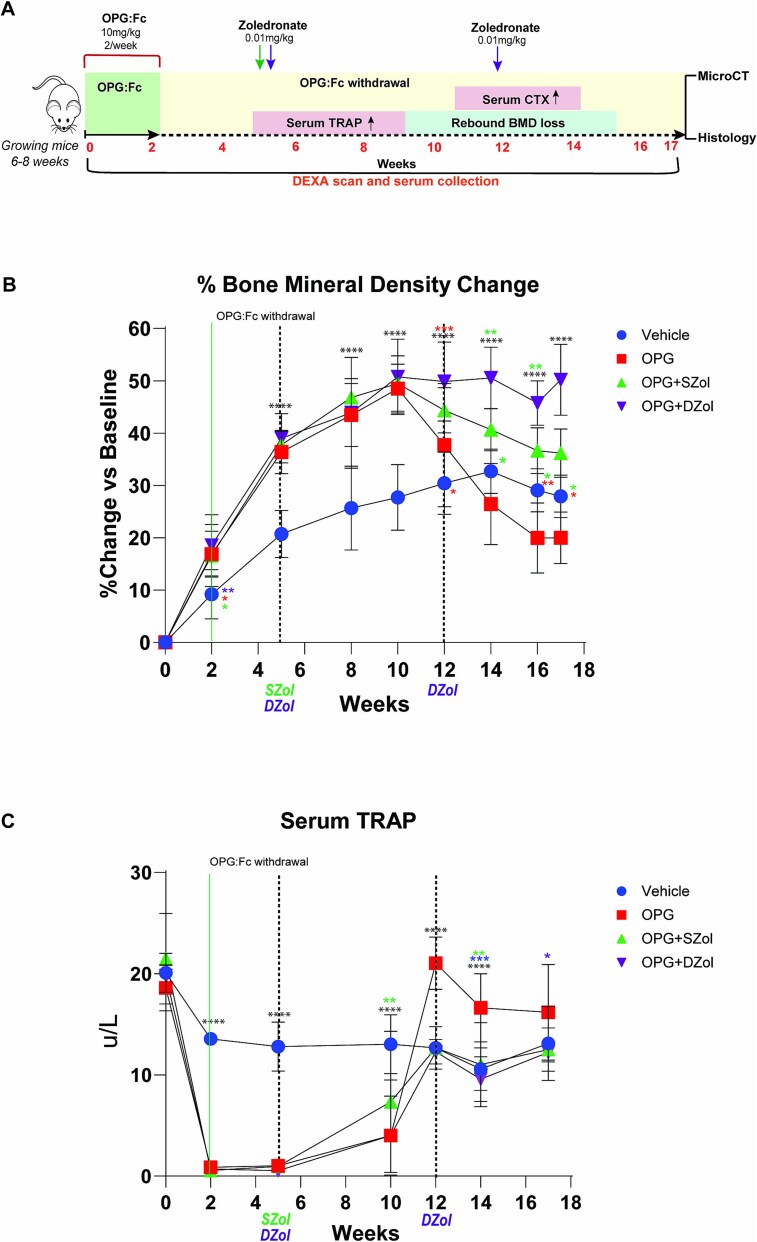
Early and multiple doses of sequential zoledronate prevents rebound BMD loss and the rise and overshoot in serum TRAP in growing mice. (A) Schematic of the experimental design to assess the effect of sequential zoledronate following OPG:Fc treatment in growing mice. Expected timing of a rise in serum TRAP, P1NP, CTX, and rebound bone loss is highlighted. (B) BMD changes following OPG:FC treatment and sequential zoledronate as early single dose at week 5 or multiple doses at weeks 5 and 12. BMD shown as a percentage change from baseline levels following treatment with OPG:Fc or saline followed by zoledronate or saline at weeks 5 and 12 (*n* = 8 per group). Data are represented as mean ± SD. The asterisks indicate *p-*values <.05 (^*^p < .05, ^**^p < .01, ^***^p < .001, ^****^p < .0001). (C) Longitudinal serum TRAP measured by ELISA at baseline and following 2 wk treatment with OPG:Fc or saline followed by zoledronate or saline at weeks 5 and 12. Data are represented as mean ± SD. The asterisks indicate *p-*values <.05 (^*^p < .05, ^**^p < .01, ^***^p < .001, ^****^p < .0001).

To examine this, mice were treated with OPG:Fc or saline for 2 wk and received zoledronate or saline at week 5, while serum TRAP remained suppressed, and a group of mice received a second dose at week 12 when rebound BMD loss was occurring to assess the effectiveness of multiple zoledronate doses (Figure 3A). Mice treated with OPG:Fc experienced BMD gains for 8 wk following treatment, peaking at week 10, 22% higher than vehicle (*p* < .0001, Figure 3B). BMD loss was observed in OPG:Fc treated mice reaching vehicle levels just 2 wk later at week 12. Mice treated with a single dose of zoledronate at week 5 experienced a 27% reduction in BMD from its peak at week 10 but levels remained significantly higher than vehicle and OPG:Fc-only mice until the end of the study at week 17. Mice that received a dose of zoledronate at both week 5 and week 12 maintained the peak BMD attained with OPG:Fc treatment and levels remained higher than all other groups until the end of the study at week 17 (*p* < .0001, Figure 3B).

Longitudinal TRAP analysis showed suppressed serum TRAP levels in mice treated with OPG:Fc at week 5 when the first zoledronate dose was administered (Figure 3C). Consistent with our model of denosumab treatment and discontinuation, a rapid rise and overshoot in serum TRAP above vehicle levels was observed in mice treated with OPG:Fc only, reaching levels 68% higher than vehicle levels at week 12 (*p* < .0001, Figure 3C). Mice that received a single dose of zoledronate at week 5 also experienced a rise in serum TRAP levels but only to vehicle levels and remained equivalent to vehicle levels until the end of the study (Figure 3C). Similarly, mice receiving zoledronate at both weeks 5 and 12 also showed a rise in serum TRAP levels to control levels which was maintained until the end of the study (Figure 3C).

### Sequential zoledronate attenuates rebound bone loss following OPG:Fc withdrawal in skeletally mature mice

**Figure 4:**
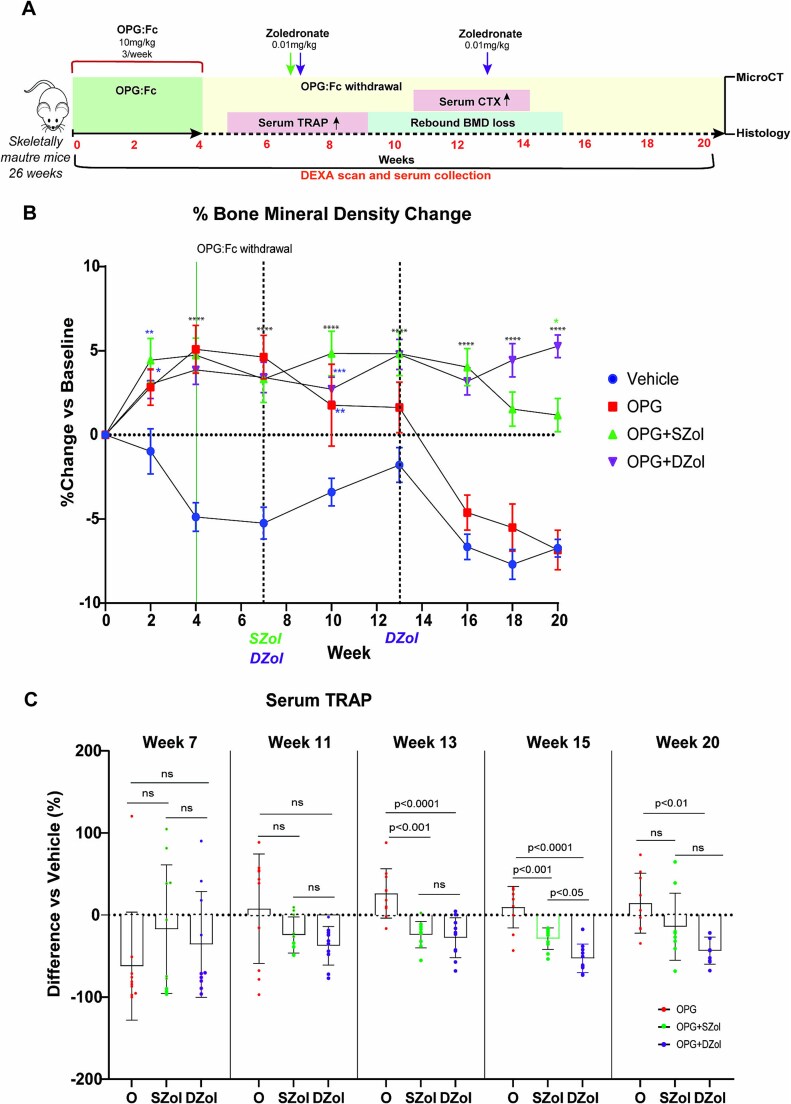
Early and multiple doses of sequential zoledronate prevents rebound BMD loss and the rise and overshoot in serum TRAP in skeletally mature mice. (A) Schematic of the experimental design to assess the effect of sequential zoledronate following OPG:Fc treatment in skeletally mature mice. Expected timing of a rise in serum TRAP, P1NP, CTX, and rebound bone loss is highlighted. (B) BMD changes following OPG:Fc treatment and sequential treatment at weeks 7 and 13. BMD is shown as a percentage change from baseline levels following treatment with OPG:Fc or saline followed by zoledronate or saline at weeks 7 and 13 (*n* = 10 per group). Data are represented as mean ± SD. The asterisks indicate *p-*values <.05 (^*^p < .05, ^**^p < .01, ^****^p < .0001). (C) Serum TRAP measured by ELISA in mice treated with OPG:Fc and sequential treatment at each timepoint compared with vehicle (*n* = 7-10 per group), expressed as a percentage difference compared with the vehicle mean. The boxplots represent mean ± SD. The displayed *p*-values indicate statistical significance between treatment groups at each timepoint.

Our model utilizes young, growing mice and therefore the effect of normal skeletal growth is an important consideration. To address this limitation and to confirm the effect of sequential zoledronate following OPG:Fc withdrawal, we treated 26-wk-old, skeletally mature mice with OPG:Fc or saline for 4 wk until there was a significant increase in BMD between treated mice compared with controls (Figure 4A). Three weeks following the end of the treatment period, mice were treated with zoledronate or saline at week 7, and a group received a second dose of zoledronate at week 13 when a significant decline in BMD was occurring (Figure 4B). BMD loss was observed in the vehicle mice throughout the study. BMD increased in all OPG:Fc treated mice, peaking at week 4 at 9.4% above vehicle levels (*p* < .0001). A decline in BMD was noted in the mice treated with OPG:Fc-only from week 7, reaching vehicle levels by week 13. Both the vehicle and OPG:Fc-only mice had lower BMD at the end of the study compared with at that start of the study (−6.7% and −6.8% from baseline, respectively). Mice that received zoledronate maintained significantly higher BMD compared with vehicle throughout the study. In the mice that received a single dose of zoledronate at week 7, BMD loss was observed from week 16, though this remained significantly higher than vehicle and OPG:Fc-only mice. Mice that received a second dose of zoledronate at week 13 maintained significantly higher BMD and reached levels higher than mice that received a single dose at the end of the study (4.3% higher vs single dose ZOL, *p* < .05) (Figure 4B).

At week 7, at the time of the first zoledronate dose, serum TRAP was below vehicle levels in most of the OPG:FC treated mice though some mice in each group had started to experience a rebound overshoot in serum TRAP above vehicle levels (Figure 4C). Four weeks later at week 11, serum TRAP levels were below vehicle levels in mice treated with zoledronate. However, in mice treated only with OPG:Fc, serum TRAP levels rose to vehicle levels with more mice experiencing an overshoot in serum TRAP compared with week 7. By week 13, serum TRAP in OPG:Fc-only mice had overshot above vehicle levels and this persisted until the end of the study. Mice treated with zoledronate maintained serum TRAP levels below vehicle at week 15, but mice treated with a single dose of zoledronate experienced a rise in serum TRAP by week 20, which was equivalent to vehicle and OPG:Fc-only mice at this timepoint. Notably, mice treated with a second dose of zoledronate at week 13 had significantly lower serum TRAP levels compared with all other groups at week 15, and this remained lower than vehicle levels at the end of the study (Figure 4C).

Taken together, these results show that even a single early dose of zoledronate was able to attenuate the rise and overshoot in serum TRAP, and the rebound BMD loss following withdrawal of RANKL inhibition. A second dose of zoledronate was able to consolidate the BMD gains made during treatment with RANKL inhibition and prevent the rebound bone loss. Importantly, the efficacy of this treatment strategy was seen in both young, growing mice and older, skeletally mature mice.

### Early sequential and multiple zoledronate therapy maintains bone gains in both trabecular and cortical compartments in the femur

The rebound phenomenon following denosumab discontinuation leads to the loss of bone gained during treatment to pretreatment levels. To examine the effect of early and multiple zoledronate treatment on consolidating bone gains made during RANKL inhibition, we harvested femora at the end of the study for ex vivo analysis with microCT.

**Figure 5:**
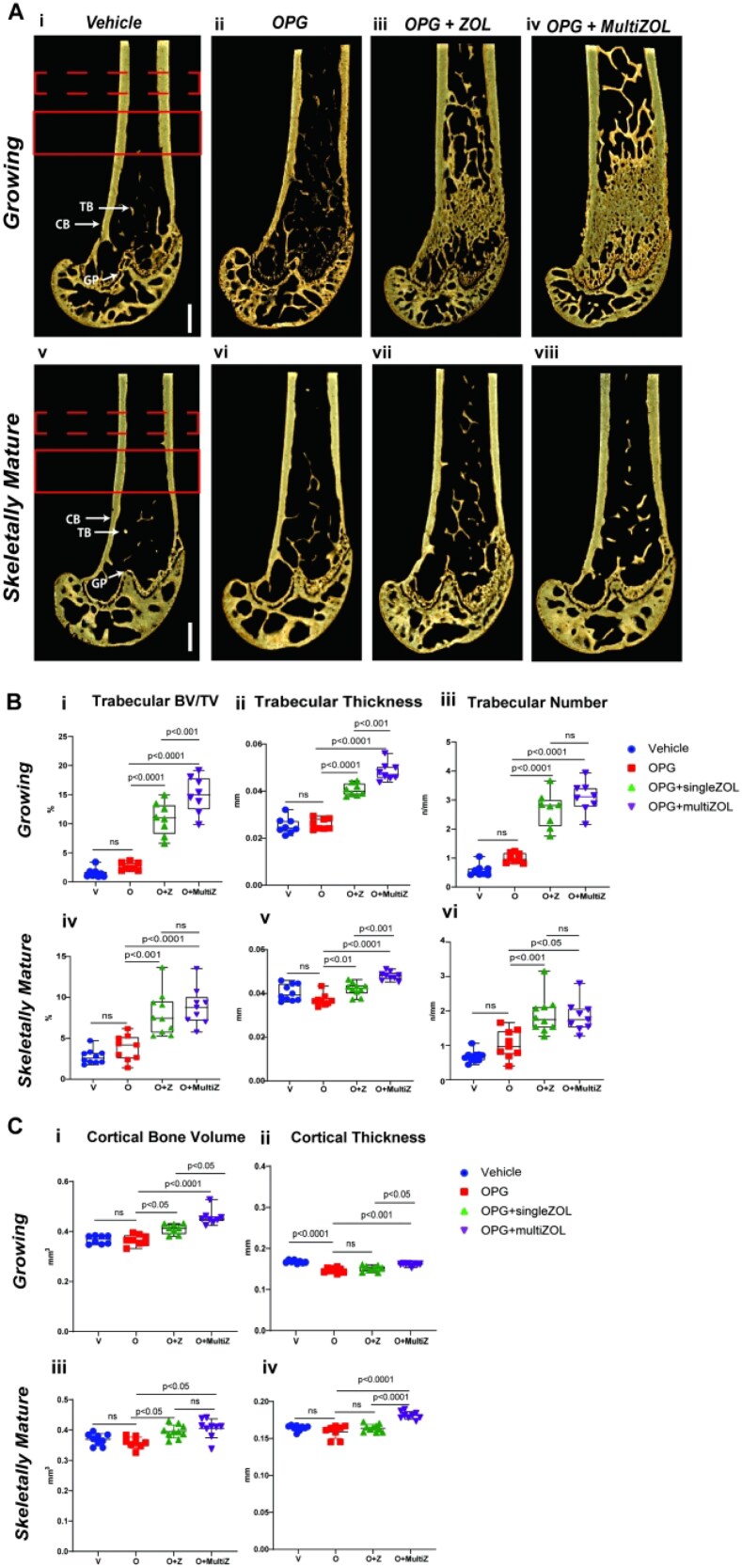
Changes in bone microarchitecture in the femur following OPG:Fc treatment and sequential zoledronate therapy. (A) Representative 3D images of harvested femora showing differences in bone microarchitecture between mice treated with saline and OPG:Fc followed by zoledronate or saline in (i-iv) growing and (v-viii) skeletally mature mice. The dashed red box denotes an ROI examined at a 0.5 mm section located 3 mm above the growth plate (GP) where the cortical parameters are calculated. The solid red box denotes ROI examined at a 1 mm section located 2 mm above the growth plate where the trabecular parameters are calculated. (B) Differences in trabecular volume (i, iv), thickness (ii, v), and number (iii, vi) between growing mice or skeletally mature mice treated with saline (vehicle) or OPG:Fc followed by sequential zoledronate. The boxplots represent mean ± SD. (C) Differences in cortical volume (i, iii) and thickness (ii, iv) between growing mice or skeletally mature mice treated with saline (vehicle) or OPG:Fc followed by sequential zoledronate. The boxplots represent mean ± SD. Abbreviations: CB, cortical bone, TB, trabecular bone.

Trabecular parameters were calculated in the distal metaphyseal region to reduce the contribution of the primary spongiosa in this analysis (Figure 5A). Comparing the femurs harvested at the end of the study following completion of rebound bone loss, there was no difference in trabecular volume (BV/TV), thickness, or number between control and mice treated with OPG:Fc in both growing and skeletally mature mice, aligning with DXA data showing the OPG:Fc group had rebounded to vehicle levels at this time. However, trabecular parameters were significantly higher in both growing and mature mice treated with zoledronate compared with vehicle and OPG:Fc treated mice that did not receive zoledronate (Figure 5B). Growing mice that received 2 doses of zoledronate had significantly higher trabecular volume (*p* < .01) and thickness (*p* < .001) compared with those that received a single dose, though the trabecular number was equivalent (Figure 5Bi-iii). In skeletally mature mice, those that received 2 doses of zoledronate had significantly higher trabecular thickness (*p* < .001) but equivalent volume and number compared with mice that received a single dose of zoledronate (Figure 5Biv-vi).

In the cortical compartment at the distal diaphysis, there was increased cortical volume in mice treated with zoledronate in both growing and skeletally mature mice compared with OPG:Fc-only and vehicle (Figure 5C). Growing mice that received 2 doses of zoledronate had significantly higher cortical volume compared with those that received a single dose (*p* < .05), but this difference was not seen in skeletally mature mice where the cortical volume was equivalent between those that received a single dose and multiple doses of zoledronate. Cortical thickness also showed different results between growing and skeletally mature mice. Growing mice treated with OPG:Fc-only or with a single dose zoledronate had significantly reduced cortical thickness compared with vehicle (*p* < .0001). Cortical thickness was significantly higher in mice treated with 2 doses of zoledronate compared with OPG:Fc-only (*p* < .0001) or single dose zoledronate (*p* < .0001), where skeletally mature mice treated with 2 doses of zoledronate had significantly higher cortical thickness compared with all other groups (Figure 5C). Endosteal and periosteal perimeters at the cortical ROI were significantly higher in all mice treated with OPG:Fc compared with vehicle (Figure S1). There were no differences in periosteal perimeter between all groups in the skeletally mature mice. Endosteal perimeters were also equivalent between groups except in skeletally mature mice that received 2 doses of zoledronate, which was significantly lower (Figure S1).

These results show that both trabecular and cortical parameters return to untreated, vehicle levels in mice that did not receive any sequential therapy following OPG:Fc withdrawal. Sequential zoledronate treatment improved both trabecular and cortical parameters compared with mice that did not receive any sequential therapy, particularly in mice given multiple doses of zoledronate.

### Early sequential zoledronate therapy increased vertebral fracture resistance in skeletally mature mice

Vertebral fractures are a significant concern in the management of patients following denosumab discontinuation. To assess how differences in bone microarchitecture alter fracture resistance following OPG:Fc treatment and sequential zoledronate, the L4 vertebrae underwent ex vivo microCT analysis and compression mechanical testing in both growing and skeletally mature mice.

**Figure 6:**
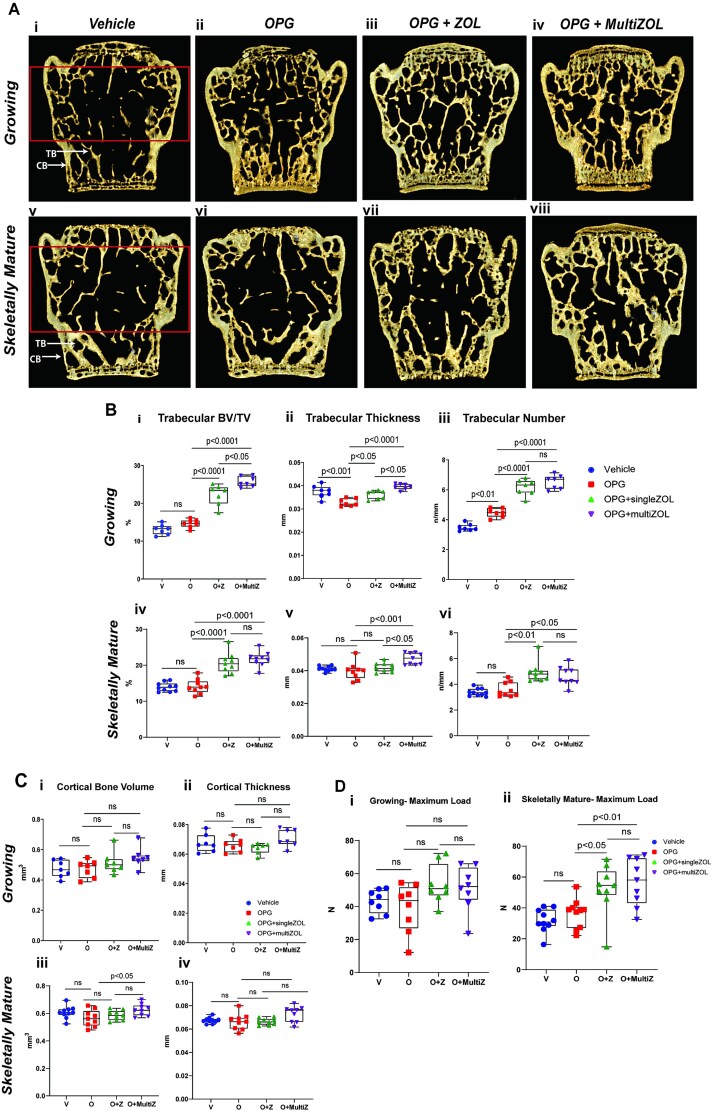
Sequential zoledronate following OPG:Fc prevented trabecular bone loss and improved fracture resistance in skeletally mature mice, but not in growing mice. (A) Representative 3D images of harvested L4 vertebrae showing differences in bone microarchitecture between mice treated with saline and OPG:Fc followed by zoledronate or saline in (i-iv) growing and (v-viii) skeletally mature mice. The solid red box denotes ROI examined at a distance between 0.2 mm offset from the point of 50% spongiosa and trabecular bone on both ends of the vertebrae. (B) Differences in trabecular volume (i, iv), thickness (ii, v), and number (iii, vi) between growing mice or skeletally mature mice treated with saline (vehicle) or OPG:Fc followed by sequential zoledronate. The boxplots represent mean ± SD. (C) Differences in cortical volume (i, iii) and thickness (ii, iv) between growing mice or skeletally mature mice treated with saline (vehicle) or OPG:Fc followed by sequential zoledronate. The boxplots represent mean ± SD. (D) Maximum load to failure (N) of L4 vertebrae from each treatment group in growing mice (i) and skeletally mature mice (ii). Boxplots represent mean ± SD. Abbreviations: CB, cortical bone, TB, trabecular bone.

Representative 3D microCT reconstructed images of the L4 vertebrae harvested at the end of the study are shown in Figure 6A. Higher trabecular volume and number were seen in growing mice that received zoledronate compared with those that did not, and this was higher in mice that received 2 doses of zoledronate. Trabecular number was equivalent in growing mice that received a single or multiple doses of zoledronate. A similar pattern was observed in skeletally mature mice though the trabecular volume was equivalent between mice that received single and 2 doses of zoledronate (Figure 6B).

There were no differences in the vertebral cortical volume or thickness between groups in growing mice (Figure 6Ci-ii). In skeletally mature mice, there was no difference in cortical thickness between groups; however, a significantly higher cortical volume was noted in mice that received 2 doses of zoledronate compared with OPG:Fc treated mice that did not receive any zoledronate (*p* < .05, Figure 6Ciii).

Despite the significant differences in the trabecular parameters, there was no difference in the maximum load to failure between groups in growing mice (Figure 6Di). In skeletally mature mice, there was a significantly higher maximum load in mice treated with either single or multiple doses of zoledronate compared with control and OPG:Fc-only groups (Figure 6Dii). There was no difference in the maximum load to failure between skeletally mature mice that received a single dose zoledronate to those that received 2 doses (Figure 6Dii) despite increased cortical bone volume seen in skeletally mature mice that received multiple doses of zoledronate compared with a single dose.

Taken together, these results show sustained increases in trabecular parameters with sequential zoledronate following OPG:Fc treatment in both growing and skeletally mature mice. However, these results translated into increased fracture resistance only in skeletally mature mice that received zoledronate.

### Abundant TRAP-positive osteoclasts are observed following sequential zoledronate, discordant with serum TRAP levels

**Figure 7:**
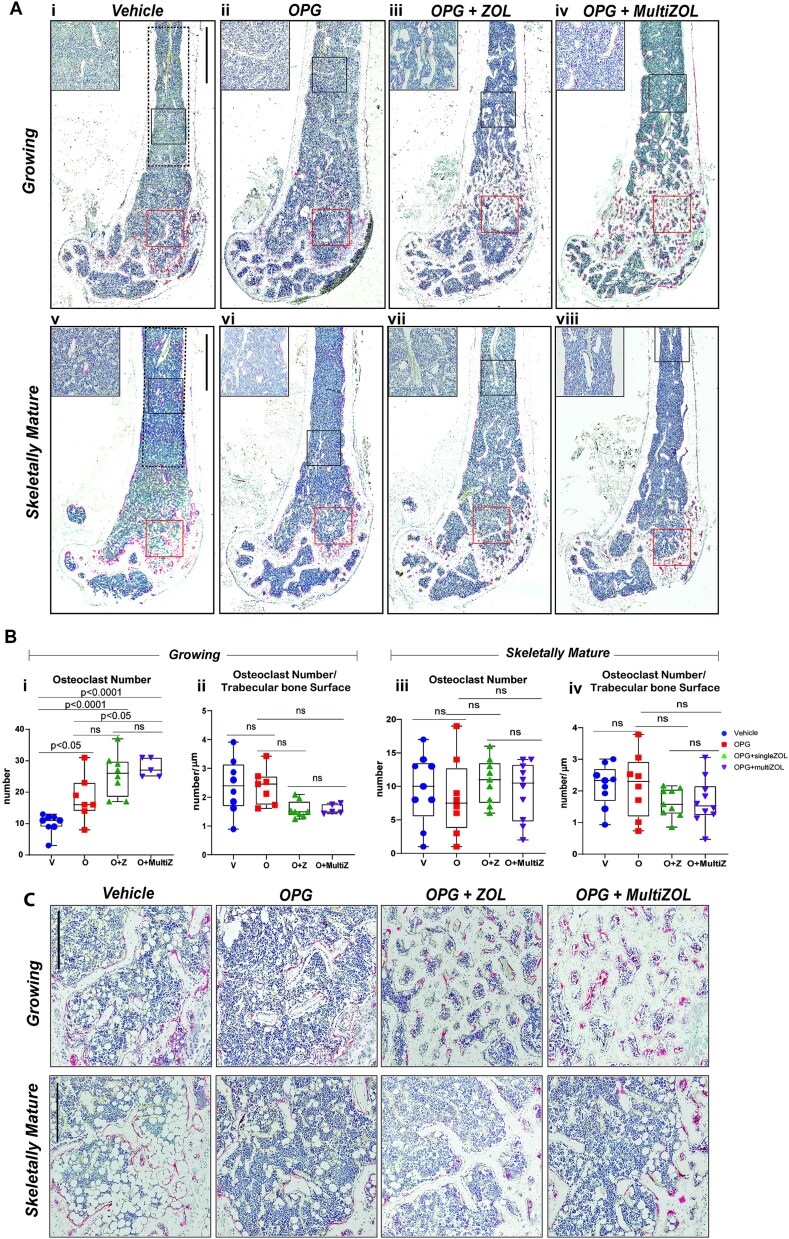
Differences in osteoclast number following OPG:Fc treatment and sequential zoledronate. (A) Representative histological images of femora stained with TRAP (red) harvested following treatment with saline or OPG:Fc. Analysis of osteoclast parameters on trabecular bone was performed within the ROI defined by the dotted line (scale bar 900 μm at 2.2x magnification). Representative high magnification images are shown in the top left corner and their corresponding ROIs are shown in the black box (scale bar 300 μm at 9.8x magnification). The red box denotes osteoclasts observed on trabecular bone surfaces as shown in magnified images in Figure 7C. (B) Quantification of number of osteoclasts (i, iii) and osteoclast surface (ii, iv) per trabecular bone surfaces in growing and skeletally mature mice. The boxplots represent mean ± SD. (C) Representative images of osteoclasts observed on trabecular bone surfaces throughout the study in the ROI marked by the red box in Figure 7A (scale bar 300 μm at 7x magnification).

To examine the differences in the number of osteoclasts following rebound bone loss and sequential zoledronate treatment, TRAP-positive cells were quantified on the trabecular bone surfaces in the distal femora at the end of the study (Figure 7A).

TRAP-positive osteoclasts were more abundant in the distal femora of growing mice treated with zoledronate compared with control (Figure 7Bi). When normalized for increased bone surface, there was a trend for fewer osteoclasts per bone surface in growing mice treated with zoledronate; however, this was not statistically significant (Figure 7Bii). There was no difference in the number of TRAP-positive osteoclasts seen in the distal femora of skeletally mature mice (Figure 7Biii-iv). No differences were seen in osteoclast surfaces between groups in both growing and skeletally mature mice (Figure S2).

While the quantification of osteoclasts was not possible in the most distal metaphyseal region of the femur, due to the retention of the primary spongiosa, TRAP-positive osteoclasts were higher in abundance in this region in growing mice that received zoledronate (Figure 7C). This difference was less pronounced in skeletally mature mice that received zoledronate.

Interestingly, higher TRAP-positive osteoclast numbers were observed despite equivalent serum TRAP between growing mice that received saline, OPG:Fc-only, or a single dose of zoledronate at the end of the studies (Figure 3C). While serum TRAP was significantly lower in mice treated with 2 doses of zoledronate, there were more TRAP-positive osteoclasts seen in growing mice that received 2 doses of zoledronate compared with OPG:Fc-only and single zoledronate treated mice. A similar pattern in serum TRAP was seen in skeletally mature mice receiving saline, OPG:Fc-only, or a single dose of zoledronate (Figure 4C) but without a difference in TRAP-positive osteoclast numbers. Skeletally mature mice that received 2 doses of zoledronate had lowest serum TRAP between the treatment groups but there was no difference in TRAP-positive osteoclast numbers.

These results show that although osteoclast number was increased in mice treated with zoledronate, when normalized to trabecular bone surface, this was no longer the case. Interestingly, differences in the number of TRAP-positive osteoclasts seen in mice treated with zoledronate did not necessarily correlate with differences in serum TRAP levels.

## Discussion

The rebound phenomenon following denosumab discontinuation is an important clinical challenge. However, an optimized sequential strategy that can consistently prevent the rapid rise and overshoot in bone resorption does not yet exist. We have previously developed and utilized an animal model of denosumab discontinuation to show that a pro-osteoclastogenic environment is present and osteoclast enzymatic activity (measured by serum TRAP) is elevated prior to the rise in serum CTX and P1NP, and serum TRAP is elevated prior to clinically detectable bone loss as measured by DXA.^18^ These results, combined with clinical studies showing bisphosphonate treatment at the time of CTX rise were inconsistent in preventing BMD loss, led us to hypothesize that earlier administration of osteoclast-directed therapy may be more effective in attenuating the rebound phenomenon, and a multiple dose strategy may be superior.

In this study, we utilized our model of denosumab discontinuation to explore the effects of early sequential and multi-dose bisphosphonate therapy and directly examine the changes in osteoclast activity and bone microarchitecture in response to this treatment strategy.

Consistent with real-world observation, administration of sequential zoledronate at a time of rise in serum CTX did not prevent rebound bone loss in our model. In addition, at the time of serum CTX rise, significant BMD loss had already occurred. We have again shown that a rise in serum TRAP from post-treatment suppressed levels precedes BMD loss and CTX rise. Further, we showed that earlier administration of zoledronate, while RANKL inhibition remained active, was able to suppress and prevent the overshoot in serum TRAP observed in our model.

Early administration of zoledronate following OPG:Fc treatment, even prior to the offset of RANKL inhibition, was able to mitigate rebound BMD loss, and an additional dose was able to prevent BMD loss, consolidating and maintaining BMD gains made on treatment in both growing and skeletally mature mice. These findings challenge the prevailing concern that early zoledronate administration would not be taken up while turnover is suppressed. Indeed, tracking uptake using fluorescent analogs in vivo, bisphosphonates have been shown to bind to so-called “quiescent” bone surfaces such as osteoclast lacunae as well as bone marrow monocytes.^23^

We utilized both growing and skeletally mature mice to allow examination of differences in the rebound phenomenon and the effects of sequential zoledronate across skeletal maturity. Growing mice receiving OPG:Fc experienced greater BMD gains compared with mature mice, reflecting contribution of normal skeletal growth. Greater BMD gains on treatment are an established risk factor for BMD loss following denosumab discontinuation.^24^ Our model previously demonstrated that longer OPG:Fc treatment, and therefore greater BMD gains, led to accelerated BMD loss.^18^ This, and increased TRAP-positive osteoclasts seen in growing mice, may explain why severe hypercalcemia is more commonly observed in pediatric patients discontinuing denosumab compared with adults.^25^ Growing bones accumulate greater bone mass with denosumab, which is then resorbed en masse by newly formed osteoclasts, releasing calcium. This aligns with observations where multiple bisphosphonate doses were required to treat post-denosumab severe hypercalcaemia in children.^26^^,^^27^

Multiple doses of zoledronate following denosumab discontinuation in adults was examined by Grassi et al.^15^ in a retrospective analysis of participants that received repeated doses of zoledronate in accordance with current recommendations, which did not prevent BMD loss and vertebral fractures. Importantly, the first dose of zoledronate was administered 7 mo following the last denosumab dose, which is likely too late to act on osteoclasts already formed and driving BMD loss. Notably, BMD remained stable in those with lower serum CTX (<280 ng/L), suggesting that zoledronate administered prior to a rise in serum CTX, and perhaps targeting the rise in serum TRAP, was able to better attenuate rebound BMD loss.

Our previous work showed that the rise and overshoot in serum TRAP accompanies rebound BMD loss, leading to our hypothesis that targeting this rise in serum TRAP with earlier intervention would mitigate BMD loss. In this work, early zoledronate administration indeed prevented the overshoot in serum TRAP and did not exceed vehicle levels in mice receiving zoledronate.

Cross-sectional analyses of serum TRAP, CTX, and RANKL at 6, 9, and 12 mo following the last denosumab dose were performed by Sølling et al.^28^ This showed significantly lower serum TRAP at 6 mo compared with 9 and 12 mo, whereas serum RANKL was significantly higher at 6 mo. The authors concluded that elevated RANKL levels most likely result in increased osteoclasts (measured by TRAP), consistent with findings in our model.^18^

Utility of serum TRAP as a marker following denosumab discontinuation was explored by Makras et al.,^29^ where serum TRAP and CTX were measured 6 mo following the last denosumab dose. The authors did not find a relationship between the duration of denosumab and serum TRAP or the TRAP:CTX ratio and concluded that serum TRAP was not a useful early marker. However, this negative finding may be due to the timing of sampling. Serum samples were collected 6 mo following the last dose, whereas our model indicates that the rise and overshoot in serum TRAP may occur earlier. Serum TRAP measurement earlier than 6 mo following the last denosumab dose, as acknowledged by the authors, and clinical studies examining the longitudinal changes in serum TRAP may provide a better assessment of its value as a marker of osteoclast activity in this context.

Interestingly in our study, there was an increased number of TRAP-positive osteoclasts throughout the femora of mice that received sequential zoledronate, discordant with differences in serum TRAP. This suggests that serum TRAP reflects the enzymatic activity of resorbing osteoclasts, rather than the number of osteoclasts present.^30^ This was particularly evident in mice that received 2 doses of zoledronate as serum TRAP was lowest despite higher or equivalent osteoclast numbers. We observed an increased number of non-attached, TRAP-positive osteoclasts in both growing and skeletally mature mice treated with zoledronate (data not shown). In a model of MRONJ, where rats were treated with zoledronate, Nagata et al.^31^ showed greater TRAP-positive multinucleated cells in the dental sockets with a significantly higher proportion of non-attached osteoclasts. These results suggest that bisphosphonate therapy impairs osteoclast activity but not the formation and activation of osteoclasts.

Patients are at increased risk of fractures, especially vertebral fractures, following denosumab discontinuation. MicroCT analysis at the end of our study showed a return to control levels in both trabecular and cortical parameters in mice that received OPG:Fc alone without sequential zoledronate, whereas mice that received zoledronate had significantly improved trabecular microarchitecture in both the femur and the vertebrae. However, when the lumbar vertebrae were subject to compression mechanical testing to assess fracture resistance, the increased trabecular parameters in zoledronate treated mice did not translate to higher maximal load to failure in young, growing mice, though a significantly higher maximal load to failure was observed in skeletally mature mice that received zoledronate. This may be due to the timing of mechanical testing as this was performed at the end of the study when rebound BMD loss was complete and there was continued skeletal growth in growing mice. Compression mechanical testing during rebound BMD loss may have revealed differences in fracture resistance between groups. Nonetheless, these differences in microCT parameters and maximal load to failure support the use of sequential zoledronate following denosumab discontinuation to improve bone microarchitecture and fracture resistance, especially in older patients.

Anabolic therapies promoting osteoblastic bone formation have expanded the therapeutic options available to improve bone mass.^32^ Despite their efficacy, it remains unclear if sequential anabolic therapy can effectively mitigate rebound bone loss following denosumab discontinuation. Several studies have examined sequential anabolic therapy using teriparatide^33^ or romosozumab^34–36^ with mixed results, and the optimal sequence and timing of anabolic therapies following denosumab discontinuation remains unclear.

Our studies were performed in eugonadal mice, whereas most patients treated for osteoporosis are postmenopausal women, and this represents a limitation in the clinical translation of our findings. However, our murine models consistently show that the processes driving the rebound phenomenon following the offset of RANKL inhibition occurs earlier than when we can detect rebound bone loss in the clinic. Therefore, clinical studies defining the temporal changes in osteoclast activity, specifically changes in serum TRAP and its association with changes in CTX and BMD, in the setting of denosumab discontinuation and sequential therapy are needed to translate the findings of our studies to clinical practice. As no effective strategy currently exists to prevent exposing patients discontinuing denosumab to potential harm, prospective clinical trials comparing earlier vs standard timing of sequential therapy would be highly informative.

Overall, our findings show that targeting osteoclasts earlier with sequential zoledronate can mitigate rebound BMD loss following the withdrawal of RANKL inhibition, and a multi-dose strategy is superior in preventing BMD loss. Future studies examining sequential therapy following denosumab discontinuation should therefore consider intervening earlier than 6 mo following the last denosumab dose and incorporate measurements of serum TRAP as a biomarker heralding imminent BMD loss.

## Supplementary Material

Figure_S1_zjaf008

Figure_S2_zjaf008

Seq_ZOL_JBMR_R1_Submission_(clean)_zjaf008

## Data Availability

All raw data files can be provided on request.
