# Supplementary figures and images for: Early and multiple doses of zoledronate mitigates rebound bone loss following withdrawal of receptor activator of nuclear factor kappa-B ligand inhibition
Source: J Bone Miner Res. 2025 Jan 23;40(3):413–27. doi: 10.1093/jbmr/zjaf008 (PMC11909728; doi:10.1093/jbmr/zjaf008)

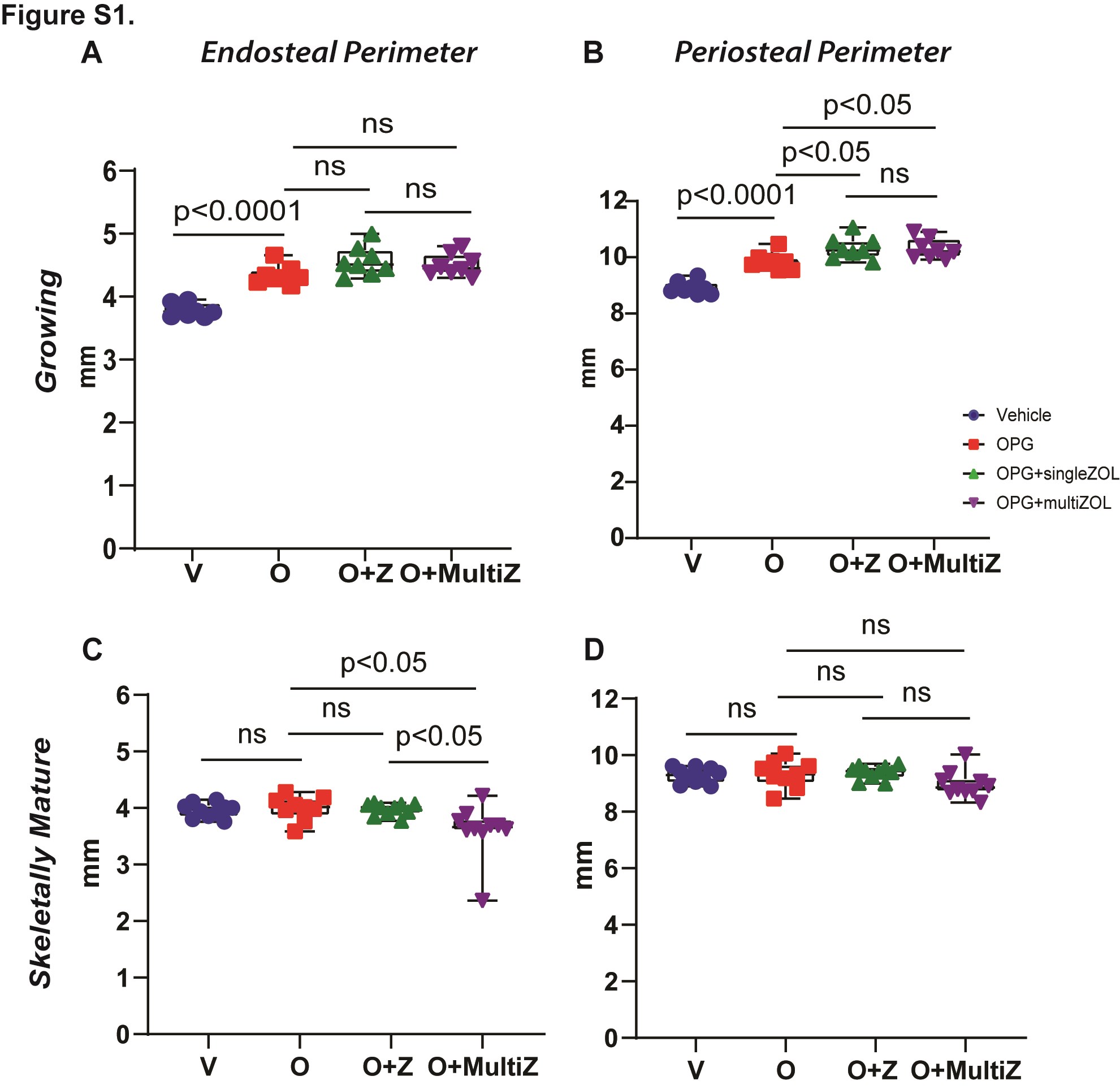

Supplement: Figure_S1_zjaf008 [file figure_s1_zjaf008.jpeg]

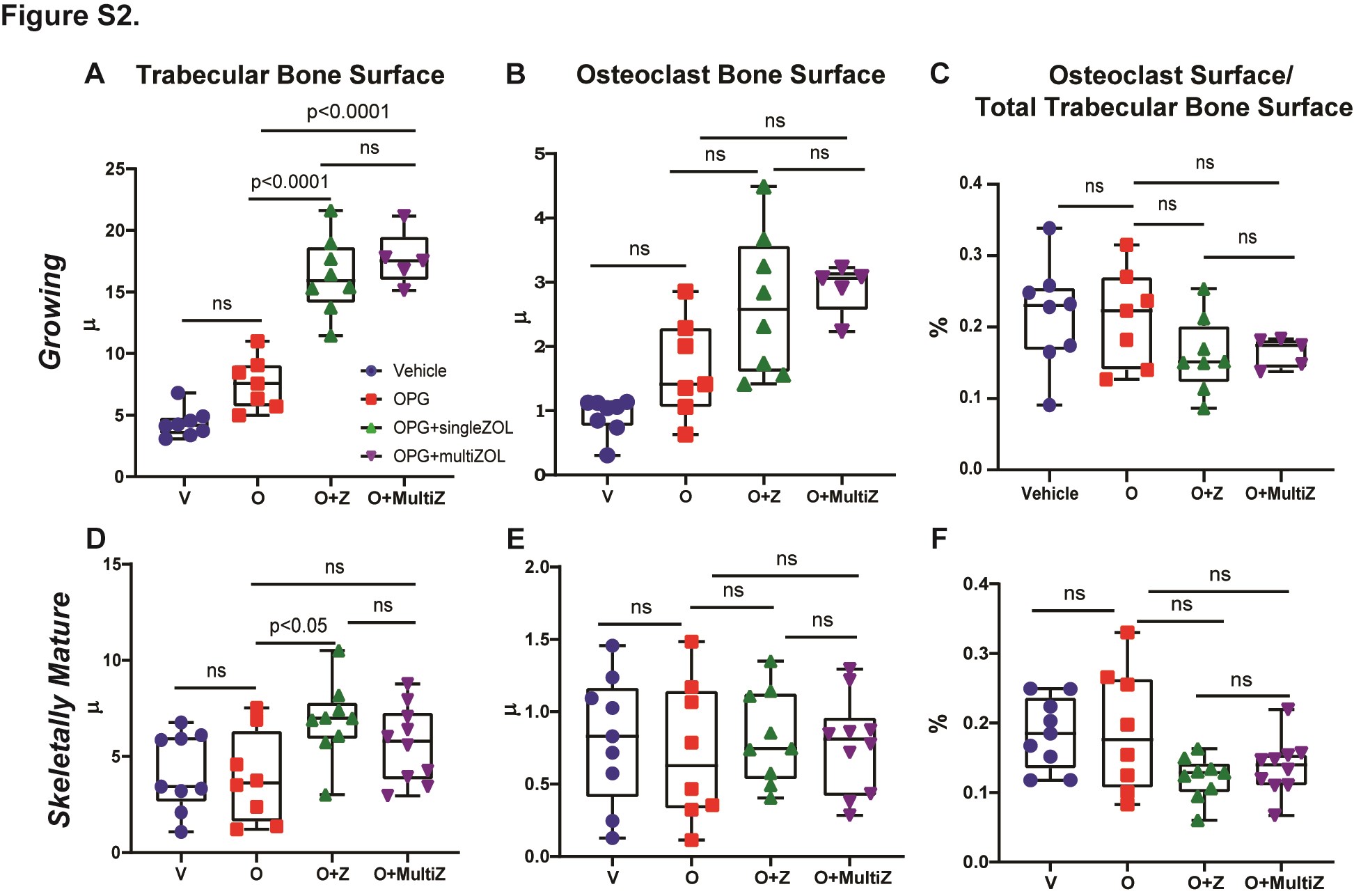

Supplement: Figure_S2_zjaf008 [file figure_s2_zjaf008.jpeg]
