# Supplementary material for: Early and multiple doses of zoledronate mitigates rebound bone loss following withdrawal of receptor activator of nuclear factor kappa-B ligand inhibition
Source: J Bone Miner Res. 2025 Jan 23;40(3):413–27. doi: 10.1093/jbmr/zjaf008 (PMC11909728; doi:10.1093/jbmr/zjaf008)
Supplement: Seq_ZOL_JBMR_R1_Submission_(clean)_zjaf008 [file seq_zol_jbmr_r1_submission_(clean)_zjaf008.docx]

**Supplementary Figure 1: Differences in femoral endosteal and periosteal perimeters in growing and skeletally mature mice treated with OPG:Fc and zoledronate**

Endosteal (A,C) and periosteal (B,D) perimeters in the femora of growing and skeletally mature mice treated with OPG:Fc and sequential zoledronate (or saline). Perimeters were calculated from the region of interest used to calculate cortical parameters, as shown by dashed red box at a 0.5mm section located 3mm above the growth plate in Figure 5A. Boxplots represent mean ± SD.

**Supplementary Figure 2: Trabecular bone surfaces are increased with sequential zoledronate treatment without an increase in osteoclast bone surfaces**

Difference in trabecular bone surface (A,D), osteoclast surface (B,E) and osteoclast surface per trabecular bone surface (C,F) in mice treated with saline (vehicle), OPG:Fc only, single dose zoledronate or multi dose zoledronate. Boxplots represent mean ± SD.
